# Successful treatment of phlegmasia cerulea dolens with percutaneous thrombectomy and catheter-directed thrombolysis: A case report

**DOI:** 10.1097/MD.0000000000031483

**Published:** 2022-11-25

**Authors:** Ching-Tang Chang, Ching-Di Chang

**Affiliations:** a Department of Emergency, Kaohsiung Municipal Ta-Tung Hospital, Kaohsiung City, Taiwan; b Department of Radiology, Kaohsiung Chang Gung Memorial Hospital, Chang Gung University College of Medicine, Kaohsiung City, Taiwan.

**Keywords:** deep vein thrombosis, limb cyanosis, limb pain, limb swelling, phlegmasia cerulea dolens

## Abstract

Phlegmasia cerulea dolens (PCD) is an uncommon condition of acute deep vein thrombosis, characterized by marked swelling of the affected limb with pain and cyanosis. High mortality and morbidity rates have been reported. Early diagnosis, quick and effective treatment are crucial for preventing potential limb loss or even death.

**Patient concerns:**

A 39-year-old woman presented to our emergency department with painful swelling and cyanotic discoloration of the left lower limb for 2 days.

**Diagnoses:**

Computed tomography revealed thrombosis in the left common iliac vein and inferior vena cava. Angiography demonstrated extensive venous occlusion from the lower inferior vena cava to the left popliteal vein. The diagnosis of PCD was made.

**Interventions:**

Systemic anticoagulation with intravenous unfractionated heparin was initiated immediately. Mechanical thrombectomy with Angiojet and angioplasty were performed, and catheter-directed thrombolysis (CDT) was administered subsequently.

**Outcomes:**

Follow-up angiography revealed regression of the thrombosis and the opacification of the deep vein was restored. The patient was discharged from the hospital uneventfully.

**Conclusions:**

PCD is a rare but potentially limb and life-threatening condition that requires immediate recognition. Treatment should be in a timely manner. Anticoagulation alone may be inadequate and more aggressive management such as CDT and thrombectomy should be considered.

## 1. Introduction

Phlegmasia cerulea dolens (PCD) is an uncommon condition of acute deep vein thrombosis (DVT), characterized by marked swelling of the affected limb with pain and cyanosis. PCD results from acute massive thrombosis in the deep venous system of the extremities, which in turn causes an obstruction of venous return. This leads to pressure change between blood vessels and adjacent tissues which ultimately causes interstitial edema and fluid sequestration in the limb. Shock develops because of plasma loss and hypovolemia. Owing to the hypotensive state and pressure increases in the interstitial tissue and compartment space, arterial system finally collapses, and gangrene ensues. High mortality and morbidity rates have been reported.^[1]^ Early diagnosis, quick and effective treatment are crucial for preventing potential limb loss or even death. Therefore, PCD is a true limb-threatening emergency that needs to be rapidly recognized and treated promptly.

## 2. Case report

A 39-year-old woman presented to our emergency department because of the left lower limb painful swelling for 2 days. There was no history of fever or trauma. She also denied chest pain, dyspnea, or abdominal pain. She had a past medical history of thyroidectomy and took Eltroxin because of hypothyroidism.

**Figure 1.**
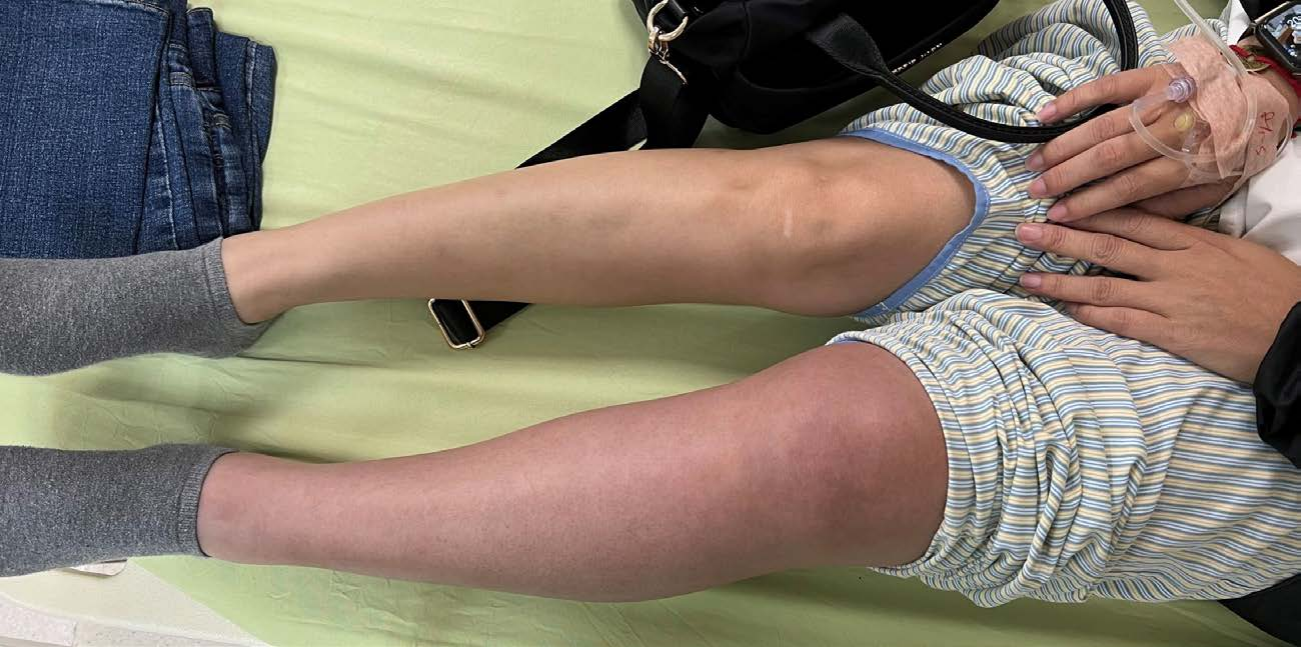
The patient’s left lower limb showed swelling with cyanotic discoloration.

**Figure 2.**
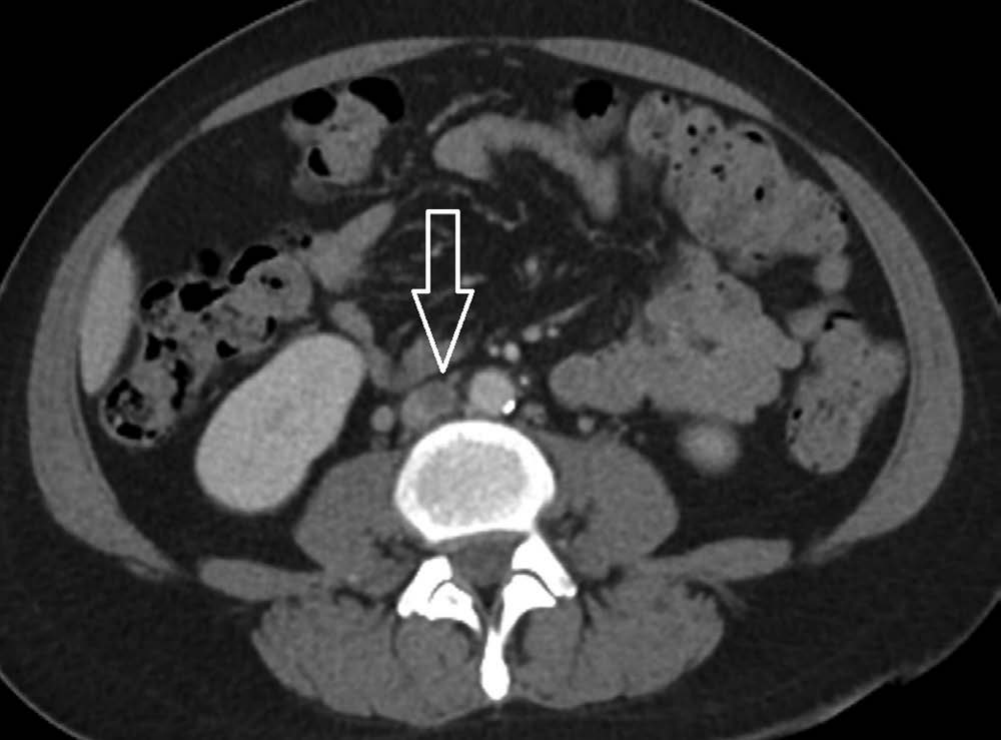
Axial image of computed tomography showing thrombosis (arrow) in the inferior vena cava.

**Figure 3.**
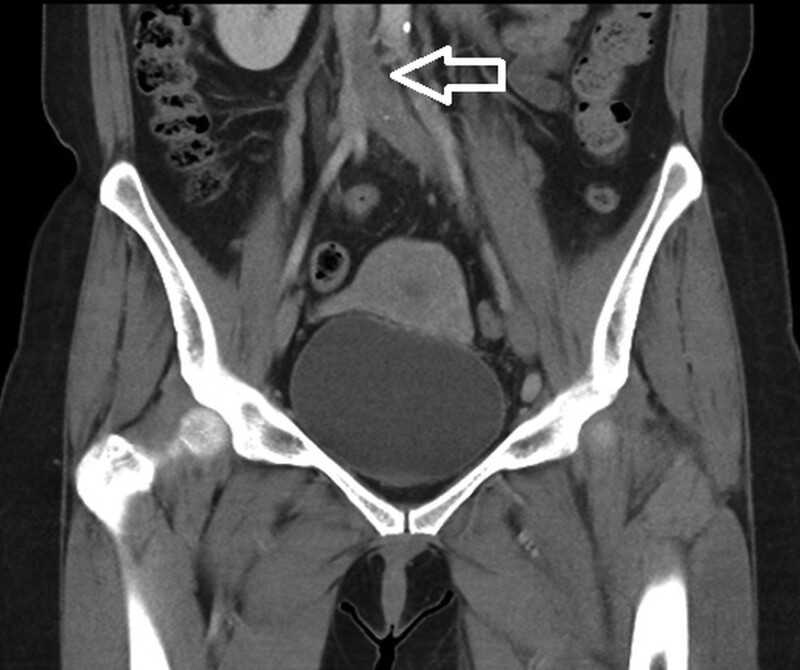
Coronal computed tomography indicating thrombosis (arrow) in the inferior vena cava and left common iliac vein.

On arrival, the patient’s vital signs were stable. The body temperature was 36.7°C. The heart rate was 98 beats per minute and the respiratory rate was 16 breaths per minute. The blood pressure was 141/91 mm Hg and SpO2 was 95% on room air. Physical examination showed swelling of the left lower limb from upper thigh to foot with cyanotic discoloration (Fig. 1). The distal pulsation from dorsalis pedis artery was palpable. Laboratory data revealed leukocytosis (WBC 19760), elevated C-reactive protein (31.43mg/L), and extreme high D-dimer (9.87 µg/mL). Computed tomography was performed under the suspicion of acute DVT. The result showed extensive thrombosis affecting the left common iliac vein and inferior vena cava (Figs. 2 and 3). Systemic anticoagulation with intravenous unfractionated heparin was initiated immediately. The patient was then transferred to a medical center for further treatment.

After being transferred to the medical center, left lower limb angiography was performed and the results showed venous occlusion from the distal inferior vena cava to left common and external iliac veins, left common and superficial femoral veins, and the left popliteal vein. Mechanical thrombectomy with Angiojet and angioplasty were performed, and catheter-directed thrombolytic therapy was administered subsequently. Follow-up angiography performed 3 days later revealed restored blood flow of the deep venous system. The anticoagulant was changed to Apixaban during the next few days, and she was discharged uneventfully from the hospital.

## 3. Discussion

PCD is a less frequently encountered form of DVT and is associated with a high incidence of morbidity and mortality. The major amputation rate is 20% to 50% and the mortality rate is 20% to 40%.^[1]^ Despite prompt treatment, a significant proportion of patients still experience venous valvular insufficiency and post-thrombotic syndrome.

Treatment is aimed at maintaining hemodynamic stable, preventing further blood clot aggregation, reducing the thrombus burden, and restoring venous outflow. Fluid resuscitation and leg elevation are important for the early treatment of PCD.^[1,2]^ Anticoagulation should be started immediately unless there is a contraindication. Under most circumstances, anticoagulation alone is insufficient for the treatment of PCD. More aggressive vascular intervention, such as catheter-directed thrombolysis (CDT) and thrombectomy (catheter-directed or surgical) are often required to help remove the massive thrombus burden.

Before the advent of endovascular therapy, surgical thrombectomy was the treatment of choice. Recently, percutaneous catheter-based techniques are the mainstay for early thrombus removal. Compared with standard anticoagulant therapy, CDT is associated with significant reductions in the risks of post-thrombotic syndrome, venous obstruction, and venous reflux.^[2,3]^

Pharmacomechanical thrombectomy has also been proven to be an effective alternative or adjunctive therapy to catheter-directed thrombolysis. When compared to CDT, lesser thrombolytic infusion dose and time, shorter intensive care unit and total hospital length of stay, and lower costs have been reported.^[2,4,5]^

In this case, the patient underwent anticoagulation therapy combined with CDT and percutaneous mechanical thrombectomy. The treatment result was satisfactory. No major complications such as limb ischemia, compartment syndrome, or hemorrhage occurred during the hospital stay. Long-term follow-up for monitoring the complications of DVT as well as those of anticoagulation therapy may be necessary.

## 4. Conclusion

PCD is a rare but potentially limb and life-threatening condition that requires immediate recognition. Treatment should be in a timely manner. Anticoagulation alone may be inadequate and more aggressive management such as CDT and thrombectomy should be considered.

## Author contributions

Ching-Tang, Chang wrote the manuscript. Ching-Di, Chang reviewed and edited the manuscript.

**Writing – original draft:** Ching-Tang Chang.

**Writing – review & editing:** Ching-Di Chang.
